# The Impact of Superfast Broadband, Tailored Booklets for Households, and Discussions With General Practitioners on Personal Electronic Health Readiness: Cluster Factorial Quasi-Randomized Control Trial

**DOI:** 10.2196/11386

**Published:** 2019-03-11

**Authors:** Philip Abbott-Garner, Janet Richardson, Ray B Jones

**Affiliations:** 1 School of Nursing and Midwifery, University of Plymouth Plymouth United Kingdom

**Keywords:** eHealth, randomized controlled trial, digital divide, broadband implementation, eHealth readiness, eHealth inequalities, tailored booklet, cluster trial

## Abstract

**Background:**

Electronic health (eHealth) may improve health outcomes, but many people remain digitally excluded. Personal readiness to use the internet for health may be limited by lack of internet infrastructure, personal skills, social support, service provision, and cost. The impact of interventions to reduce these barriers is unknown. From 2011, the British Government supported the implementation of “superfast” broadband (*Superfast*) across the rural county of Cornwall. This provided the opportunity to assess the impact of interventions at regional, practice, and household levels.

**Objective:**

This study aimed to assess the impact of 3 interventions on personal eHealth readiness: (1) regional-level implementation of Superfast, (2) practice-level discussions with general practitioners to encourage greater internet use in health service provision, and (3) household-level tailored booklets providing information to help improve personal skills in eHealth.

**Methods:**

This was a cluster quasi-randomized factorial controlled trial. Implementation of *Superfast* was monitored, and postcodes were classified as having early or late availability. An algorithm selected 78 from 16,385 eligible postcodes to minimize the possibility of overlap between general practices and ensure a balance of urban and rural areas; 1388 households were randomly selected from the 78 postcodes and allocated to the 8 (2 × 2 × 2) study arms. A modified version of the Personal eHealth Readiness Questionnaire was used to compare scores (0 to 10) and 4 components (personal, provision, support, and economic) from baseline (August 2013) to the 18-month follow-up between the 8 arms, to assess the impact of interventions. We compared SDs of scores to assess changes in eHealth inequalities.

**Results:**

eHealth readiness improved over 18 months from 4.36 out of 10 to 4.59 out of 10 (*t*_235_=4.18; *P*<.001; CI=0.13 to 0.35), resulting from increases in personal and provision components of the score (*t*_255_=3.191; *P*=.002 and *t*_258_=3.410; *P*=.001). However, there were no significant differences between the 3 interventions, either singly or in combination using intention-to-treat analysis. The proportion of internet users did not significantly increase (79.2%, 205/259 to 81.5%, 211/259) and mobile use was significantly greater (50.5%, 101/199 to 64.8%, 129/199). There was no change in eHealth inequality.

**Conclusions:**

People in Cornwall became more ready to adopt eHealth services, increasing both their personal ability to use eHealth and their methods of access. The implementation of *Superfast* may have contributed to this; we are certain that our other 2 interventions did not. This increased eHealth readiness did not cause a larger digital divide. The study illustrates the complexity of conducting a randomized controlled trial to assess the impact of interventions at regional, practice, and household levels. Our method may be of use to others.

**Trial Registration:**

ClinicalTrials.gov NCT00102401; https://clinicaltrials.gov/ct2/show/NCT02355808 (Archived by WebCite at http://www.webcitation.org/75oEz0E1x)

## Introduction

### Setting

Cornwall, a county in south west England, is a rural area with a population density of 1.5 persons per hectare versus the average of 4.1 persons per hectare in England [1]. It has a dispersed settlement pattern of numerous towns, villages, and hamlets; 27% of the population lives in urban areas, 29% in towns and larger villages, and 44% elsewhere [2]. Access to health care, transport, employment, information and communication technology (ICT), training, community facilities, and services such as shops and schools is a problem. Cornwall’s population is older than the national average (29.7% aged older than 60 years compared with 22.3% nationally [1]). Before the Superfast Cornwall project (discussed below), internet infrastructure was poor. Maximum download speed averaged 5 to 6 Mbps in urban areas [3], and some *not spot areas* had no internet access. Internet reliability was poor, meaning access could often fluctuate during the day.

### Superfast Cornwall Project

This program funded by the European Union, British Telecom (BT), and Cornwall Council aimed to provide superfast broadband (*Superfast)* infrastructure to Cornwall and the Isles of Scilly. The program ran from 2011 to 2015, during this duration, fiber optic broadband had been introduced to 95% of homes and businesses [3]. *Superfast*, defined as an infrastructure capable of delivering internet speeds higher than 24 Mbps [4], aimed to provide a faster and more reliable service with speeds of up to 330 Mbps. Introducing *Superfast* was a significant engineering task costing approximately £132 million and requiring the installation of 130,000 km of fiber optic cable [3].

### Benefits of Electronic Health and Digital Divide

Systematic reviews have shown the potential positive impact of electronic health (eHealth) [5,6] in areas such as the management of long-term conditions [7-11], internet-delivered cognitive behavioral therapy [12-14], smoking cessation [15,16], and cost reductions [17-22]. Some are concerned that as we introduce more eHealth, the *digital divide* will increase. Although the proportion of nonusers had declined from 35% in 2003, 22% of the British population had still not used the internet in 2013 [5]. Age remained the biggest predictor of nonuse; in 2011, only 33% of those aged 65 years and older in the United Kingdom used the internet. People who stay offline have reduced opportunities [23], and this divide could increase with the implementation of *Digital First* across the National Health Service [24].

### Barriers to Electronic Health

Differential access to information and computer technologies can be examined at the personal level [25], categorizing barriers as (1) provision (including the impact of lack of suitable infrastructure), (2) personal, (3) interpersonal, and (4) economic.

#### Provision Barrier

Poor internet access is a barrier to eHealth use [26-29]; in 2014, the average broadband speed in some rural areas was 5 Mbps compared with 27 Mbps in urban areas [30]. Slow-speed internet obviously compromises viewing of Web-based videos and images [31,32]. Variation is not just caused by hardware but can result from differences by geography or patient group in NHS services; for example, most renal patients in the United Kingdom have had access to their Web-based renal medical record for many years [33] but few, if any stroke patients had such access [34]. Video consultations had been used for dermatology [22,35] but not widely adopted in general practice.

#### Personal Barrier

Physical and psychological attributes can also be barriers, such as lack of ICT skills [36-39], distrust of internet [40] or health information it provides [41-43], and lack of motivation to access eHealth services [44,45]. Someone’s current health may increase motivation to use the internet for health information [46,47], even as it limits their ability to do so [48]. Although video use is increasing, much internet health information is text based, meaning low-literacy populations can struggle to use information effectively [26,28,49,50].

#### Interpersonal (Social) Barrier

Some factors limiting eHealth use may be moderated if people have social support [34]. Many nonusers have some form of indirect access to the internet via other individuals (proxy users). In the United Kingdom, in 2013, approximately 70% of nonusers reported having access to a proxy user but only 20% actually used them to access the internet [51]. Nonusers who do not have access to or choose not to use the internet may lack a strong support structure to help them overcome fears and apprehension [52]. With decreased social connection, some may also lack exposure to the internet and other technologies [53]. Furthermore, they may not perceive the usefulness in adopting internet use or have limited motivation to do so [54,55].

#### Economic Barrier

UK national figures indicated that lower-income households were less likely to access the internet [56]. Although homes may be technically capable of internet connection, families may not be able to afford it; someone relying on accessing the internet at their local library may be restricted by transport costs [34]. Women diagnosed with breast cancer were less likely to use the internet for health if they had a lower income, even after controlling for other predictors [57]. Lung cancer patients with higher income were more likely to seek Web-based health information about their condition [58].

### Measuring Electronic Health Readiness

The degree to which people are prepared and able to use eHealth can be termed “eHealth readiness” [59]. eHealth readiness has been approached in various ways with some focusing on the readiness of a whole sector or system; Legare et al [60] identified 6 different assessment tools [61-66] for this approach. Others have assessed the eHealth literacy of individuals, for example, the eHealth literacy scale (eHEALS) [67]. Jones [34] took a pragmatic compromise examining eHealth readiness of individuals, but including in this their opportunities from infrastructure, economics, and social support. The Personal eHealth Readiness Questionnaire (PERQ) [68] was designed to measure the impact of interventions that aimed to improve eHealth readiness and reduce eHealth inequalities. PERQ uses a similar approach to eHEALS, adopting the use of scales as opposed to a binary measure, but included further variables to cover the full range of individuals from noninternet users to frequent internet users. PERQ has 4 subcomponents: provision, personal, support, and economic.

### Measuring Electronic Health Inequalities

As older people have lower use of ICT, some observers assume that the digital divide will disappear with newer generations [29,69]. However, reduced ability to adopt new technology with age may continue [29]. Economic barriers may remain if ICT costs are too high for future generations of older adults. In addition to the ethical argument for addressing eHealth inequalities, such inequalities make the adoption of more cost-effective health delivery difficult if both eHealth and more traditional services must be provided [34]. We need, therefore, to develop interventions that help reduce eHealth inequalities and have a way of measuring them. The SD of the PERQ eHealth readiness score provides a measure of eHealth inequalities.

### Assessing the Impact of Superfast on Electronic Health Readiness

Although poor internet infrastructure is recognized as a barrier to eHealth, there was no clear evidence that improving internet infrastructure alone is enough to improve uptake of eHealth services. A simple before-after comparison does not allow the attribution of likely improvement to the infrastructure change without some form of *control group*. However, many would argue that an infrastructure change on its own is unlikely to radically improve uptake of eHealth but that some form of education, awareness raising, behavioral and organizational change is also needed. The implementation of *Superfast* provided an opportunity to assess the impact of an improved internet infrastructure alongside interventions at practice and household levels.

### Assessing the Impact of a Combination of Interventions on Electronic Health Readiness

Individuals possess different levels of eHealth readiness and, as discussed, may experience a wide range of separate and shared barriers. Barriers to the implementation of eHealth exist at multiple levels: (1) individual level, (2) clinician or service level, and (3) regional infrastructure level. It is unlikely that a single standardized intervention will be effective across these levels. Rather, it is likely that a combination of interventions, targeted at multiple levels, would prove most effective at reducing eHealth inequalities.

The *Superfast* project not only allowed for the impact assessment of an infrastructural change but also provided the opportunity to identify and assess the effectiveness of other interventions targeted at the personal and service levels. These interventions were designed to increase eHealth use, both singly and in combination. With limited resources, we sought to assess the impact of this infrastructure change in combination with individual- and provider-level interventions.

## Methods

### Design

A cluster, quasi-randomized, factorial (2 × 2 × 2) controlled trial design was used to examine the impact of 3 interventions: (1) regional-level improvement of physical infrastructure (*Superfast*), (2) practice-level discussions with general practitioners (GPs) to encourage greater use of the internet in health service provision, and (3) household-level tailored booklets (TBs) providing information to help improve personal skills in eHealth. Households within Cornwall were allocated to each of the 8 arms of the study. eHealth readiness and inequality were compared pre-and postintervention to measure the impact (singly and in combination) of each of the 3 interventions.

The study was approved by the Plymouth University Faculty of Health and Human Sciences Ethical Committee and obtained local research and development approval from the Royal Cornwall Shared Research Management Service. The trial was registered at the US National Institutes of Health (ClinicalTrials.gov) # NCT02355808 on April 2, 2015.

### Sampling and Randomization

The initial sampling unit was the postcode. All 20,088 postcodes in Cornwall (excluding the Isles of Scilly for practical reasons) were included; 2958 listed as having a population of zero and postcodes without any population data were excluded. To more clearly define the presence or absence of *Superfast,* we excluded 745 postcodes with *Superfast* coverage of between 0% and 49% as these were in the process of receiving *Superfast* at the time of sampling. The remaining 16,385 postcodes, therefore, either had *Superfast* available or did not.

Providing an intervention at the primary care level via GP practices introduced the likelihood of contamination between intervention groups. GP practices often serve a large geographical area; any intervention at this level would affect several postcode clusters. This meant that random selection of postcodes, without accounting for the intervention area, would likely allocate postcodes with shared practices to separate intervention groups. The sampling method sought to reduce the likelihood of contamination by eliminating postcode clusters at the practice level.

GP practices were included based on longitude and latitude data from NHS choices [70]. GPs in Cornwall and those in Devon on the Cornish border, who were the closest GP to a Cornish postcode, were included.

We designed a method to (1) reduce potential contamination between the 8 arms of the study, (2) account for the rollout of *Superfast*, and (3) ensure similar allocation of urban and rural areas.

**Figure 1:**
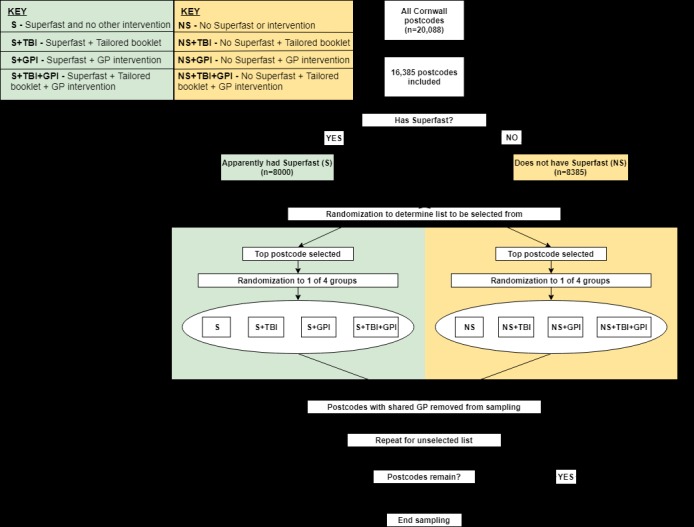
Sampling and randomization method needed to identify and randomly allocate general practice intervention within Superfast intervention. GP: general practitioner.

The 16,385 postcodes that met the initial inclusion criteria were allocated to 2 separate lists based on their *Superfast* coverage and sorted based on population (highest population at top):

- Has *Superfast* (S): postcodes with coverage ≥50% (n=8000)
- Did not have *Superfast* (NS): postcodes with coverage of 0% (n=8385).

Postcodes within these lists were randomized to intervention groups:

- No further intervention
- General practice intervention (GPI)
- Tailored booklet intervention (TBI)
- Tailored Booklet and GPI (TB+GPI).

The following process of selection and randomization (using Excel random number generation) then took place until no postcodes remained (Figure 1):

1. A randomization took place to identify which list (S, NS) would be selected first.
2. The first postcode (highest population) from the list (S, NS) was selected and was randomly allocated to either the one of 4 groups (S, S+TBI, S+GPI, S+TBI+GPI) or to the other 4 groups (NS, NS+TBI, NS+GPI, NS+TBI+GPI). There were 8 groups in total for 3 interventions (2 × 2 × 2).
3. Any postcode that shared the same geographically closest GP practice as the selected postcode was then eliminated.
4. The remaining top postcode on the second list was then selected and randomly allocated to 1 of the 4 groups.
5. This process was repeated from step 1 until no postcodes remained on either list.

Through this process, 78 from 16,385 postcodes were selected and randomly allocated to 1 of the 4 intervention groups within their level of *Superfast* coverage. Using Zoopla [68], a website giving estimated house values across the United Kingdom, all households within the postcode were listed and 18 randomly selected, using Excel number generation, and included within the study. In postcodes with less than 18 households, all households were included in the sample. The final sample consisted of 1388 households from 78 postcodes served by 78 different GP practices.

### Sample Power

Most limitations of sample size were imposed by the infrastructure intervention (Superfast arm), which was limited to the county of Cornwall. This then limited the number of GP practices that could be allocated without *contamination* between the randomization arms. The number of households found in rural postcodes limited the number of households. A sample size calculation based on the desired magnitude of effect was, therefore, not conducted. Instead, a calculation was made to estimate the possible magnitude of effect that could be found with 80% power.

With an assumed response rate of 50%, it was estimated that the smallest effect size that could be found between the 2 arms of *Superfast* (“has” and “does not have”) was 0.52, assuming 80% power and 95% significance. The smallest effect size that could be found between each of the 8 arms of the study was 1.05, with 80% power and 95% significance.

### Outcome Measures

A before versus after assessment of eHealth readiness using the PERQ [34] was conducted on households within the sample over an 18-month period. The PERQ (Multimedia Appendix 1) was modified slightly by improving the wording and layout based on recommendations in the original paper [34]. The 4 subcomponents were combined to create an overall eHealth readiness score (0 to 9). The SD of readiness scores was taken to represent eHealth inequality.

### Interventions

#### Regional: Implementation of Superfast (Had Superfast/Did Not Have Superfast)

Before the implementation of *Superfast,* households were likely to have had internet connectivity ranging from none (not-spots) to maximum speeds of 5 to 6 Mbps. After implementation of Superfast, Cornwall reported that 95% (241,000) premises had *Superfast*, with nearly 90% able to connect at speeds of over 24 Mbps [3]. It was not possible for the study to allocate postcodes to receive or not receive *Superfast*. This process was dependent on the Superfast Cornwall timescale for the rollout; therefore, this arm of the study was a *natural experiment*. Clusters were categorized into areas with or without *Superfast*, based on the rollout at the time of sampling.

#### Practice: General Practice Intervention

The aim of GPI was to engage selected practices to encourage GPs (1) to adopt more eHealth services and (2) to actively promote the existing services to their patients and aid them in adopting such services. The hypothesis was that achieving these outcomes should impact patients within the area, resulting in increased eHealth readiness. With this intervention:

1. The researcher contacted (by post) selected practices in September to October 2014 to arrange meetings. This letter explained the project and sought permission to attend practice meetings to discuss their use of eHealth services. If this was not possible, the researcher tried to meet with a practice member or establish an email conversation.
2. GPs were given suggestions as to how they might expand their current use of eHealth services to use additional eHealth services or better promote their existing services, using examples of GPs in their area or nationally.
3. GPs were also asked to comment on the services they offered, perceived benefit or detriment, and ease of adoption.

Meetings were conducted for 15 min and covered 6 topics: Web-based appointment booking; Web-based repeat prescriptions, Web-based access to medical records, information prescription, phone triage, and video consultations. Meetings were tailored to consider the current services provided by the GP practice; if discussed services were currently implemented, the conversation would focus on the difficulty the GP experienced to implement and any perceived benefits or limitations of the system. We included 39 GP practices in the GPI arm.

#### Household: Tailored Booklet

Participants randomized to the booklet intervention received a tailored eHealth information booklet by post. A total of 16 A5 pages were created using information from national and local services. Some pages were included for all participants; other pages were based on responses to the PERQ. Creation of TBs used a decision tree to identify which A5 pages to include (Multimedia Appendix 2).

This booklet was addressed to the individual who completed and returned the survey. This process identified individual needs and then tailored a booklet to address those needs. For example, a noninternet user reporting that they would use the internet more for health if they could get someone to help them received a booklet showing resources such as UK Web-based centers. On the other hand, someone who reported that they lacked confidence in using the internet received information about Web-based internet training, such as Learn My Way [71].

As a cluster trial, all households in the intervention postcodes (clusters) received an eHealth information booklet; those households not randomly selected to complete the PERQ received a general rather than personalized booklet. Tailoring for these households could only use geographical data, for example, showing a person what is available in their area based on their postcode. In this case, booklets were addressed to the household as opposed to an individual.

### Data Analysis

Data were entered into IBM SPSS version 23 for analysis. The main dependent variable (primary research question) under investigation was eHealth readiness in the form of a continuous variable, calculated from PERQ responses. Analysis was conducted using parametric tests to analyze eHealth readiness and the 4 subvariables that contributed to its calculation. On matched data, paired *t* tests were used to compare baseline with follow-up. To examine differences between groups, independent *t* tests were conducted on the change of continuous variables. Finally, a univariate general linear model was used to investigate the main effect of the 3 intervention conditions, added as fixed effects, on the change in eHealth readiness.

Some secondary analyses were conducted. The PERQ contained several categorical response questions that were relevant to the study and provided insight into eHealth behavior. For categorical data, nonparametric tests in the form of chi-squared tests for independent samples and McNemar, for paired data, were conducted. To provide further insight, in some cases, additional categorical variables were created from continuous variables to analyze proportions, for example, increased, decreased, no change.

## Results

### Response Rate

**Figure 2:**
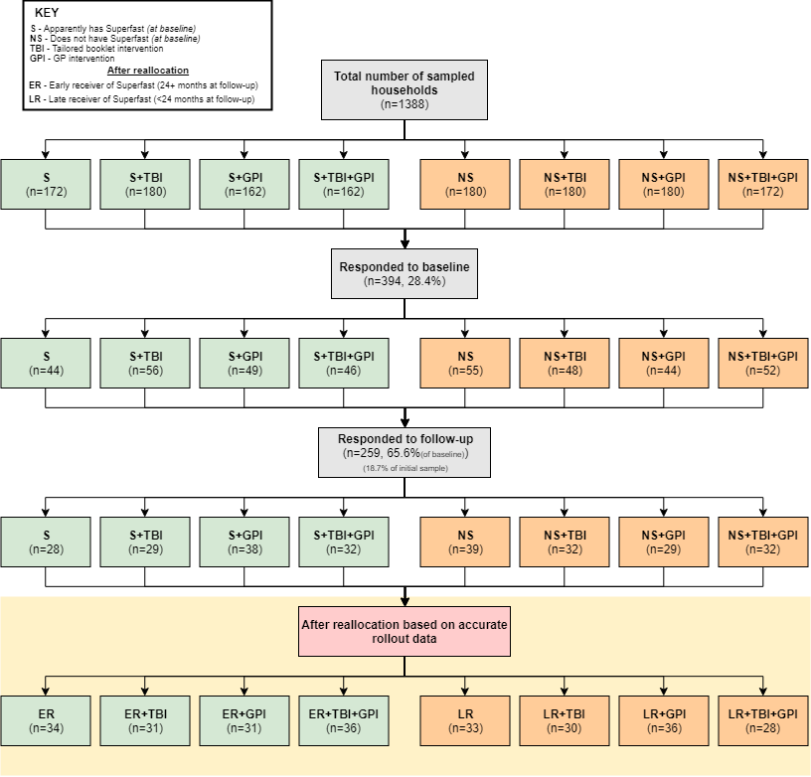
Consolidated Standards of Reporting Trials diagram of trial numbers for matched households showing early and late receivers of Superfast.

Of the 1388 households surveyed, 394 (28.4%) responded to the baseline PERQ (October 2013). At follow-up (March 2015), 259 households, 65.6% of original responders, replied to the PERQ (Figure 2).

### Regional: Implementation of Superfast

With the Superfast Cornwall project completed, more accurate rollout data were released to the researcher. These data contained precise *go live* dates for all clusters (postcodes) included in the study, allowing households to be categorized by the number of months *Superfast* had been available in their area. Households in areas where *Superfast* had been available for more than 24 months at follow-up were categorized as “early receivers.” Areas that had *Superfast* for 23 or less months at follow-up were categorized as “late receivers.”

There was no significant difference between the change in readiness (0.26 vs 0.21; *P=*.66) or provision scores (0.16 vs 0.23; *P=*.53) or between the proportion of households increasing in readiness scores between early and late receivers.

There was no difference in the perception of speed within households between baseline and follow-up (McNemar=2.46; *P*=.25). However, changes in speed perception did significantly differ between *Superfast* arms, with 12 households (14.8%, 12/81) from “late receivers” and 5 (5.2%, 5/96) from “early receivers” reporting faster internet (χ^2^_1_=4.7, *P*=.03).

### Practice: General Practice Intervention

Of the 38 GPs contacted to take part in the study, 8 (21%) agreed to take part, 3 (8%) refused due to busy schedules, and the majority (71%, 27/38) did not respond. The researcher attended 5 face-to-face meetings and had email correspondence with the remaining 3 GPs.

The GPI had no effect on household eHealth readiness, neither when considered as mean score (mean=0.18 vs mean=0.29; *t*_234_=−1.01; *P* *=*.31; CI=−0.34 to 0.11) nor when considered as proportion of households increasing in readiness scores (32.5%, 38/117 vs 34.5%, 41/119; (χ^2^_2_=.6, *P*=.74).

Overall, 18.7% (38/203) of respondents across all arms, had been given information to help them use the internet for their health by a nurse, doctor, or another health care professional, but there was no difference between those in the GPI arm and others (18.3%, 19/104 vs 19.2%, 19/99; (χ^2^_1_=.03, *P*=.87).

As many GP practices did not take part in the study, we did an *as treated* analysis comparing households from GPs who had agreed to the intervention with other households, but there was still no difference. We also counted the number of practices offering Web-based access to medical records. Only 6 GPs within Cornwall had started to offer Web-based access to medical records at follow-up, previously none had offered this facility; however, there was no difference between those in the GPI versus others.

### Household: Tailored Booklet Intervention

There was no significant effect of the booklet intervention on the change of readiness scores (*t*_234_=−.106; *P=*.92). The proportion of households increasing in readiness scores was 33.5% (79/236) overall with no difference between those receiving (36.5%, 42/115) and not receiving the booklet intervention (37/121, 30.6%; (χ^2^_2_=1.2, *P*=.56).

The PERQ calculates a separate skill score based on responders’ reported self-ability to complete 6 internet-related tasks. Overall, 32% (82/259) showed an increase in skills scores, but there was no difference between those who received a booklet compared with nonreceivers (33.6%, 42/125 vs 30.6%, 41/134; (χ^2^_2_=1.6, *P*=.46).

One area of the booklet focused specifically on the eHealth services offered by local GPs’ websites to attempt to increase knowledge and use of these services. At baseline, a total of 54 (54/204, 26.5%) households reported that they “Didn’t Know” if their local GP had a website; of these, 27 had become aware of their local GPs’ website and the services it offered, but there was no difference between those receiving or not receiving the booklet (51.9%, 14/27 vs 48.1%, 13/27; χ^2^=0.09, df=2; *P=*.96; χ^2^_2_=.09, *P*=.96).

Only 5 internet-using households (5.2%, 5/97) who had received the booklet acknowledged receiving “a booklet in the post regarding using the internet for health.”

### Interventions in Combination

A univariate general linear model was used to investigate the main and combined effect of the 3 interventions (*Superfast*, GP, booklet), added as fixed effects, on the change in eHealth readiness. A full-factorial interaction effect was also examined between *Superfast* × booklet × GP for the outcome of change in readiness. The model showed no significant main effect of either *Superfast* (*P*=.677), GP (*P*=.237), or booklet (*P*=.928) on the change in readiness scores.

### Change in Internet Use

The proportion of internet users (79.2%, 205/259) at baseline did not significantly increase, being 81.5% (211/259) at 18-month follow-up. A fifth of respondents who reported that they had not used the internet at baseline (20.4%, 11/54) reported that they had used the internet at follow-up. Only 5 internet users at baseline (2.4%, 5/205) reported not to have used the internet in the previous 3 months at follow-up.

**Figure 3:**
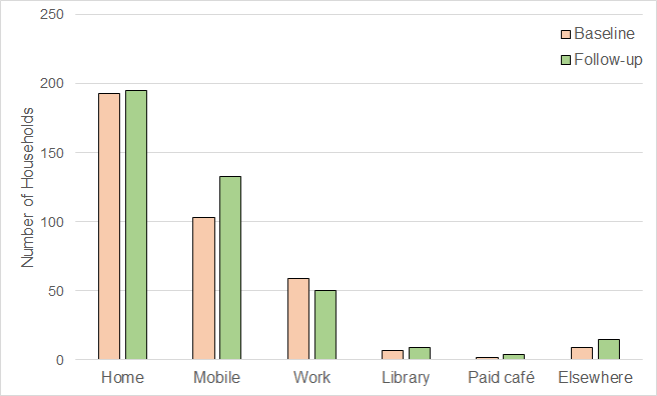
Where and how 211 internet-using households had accessed the internet in the last 3 months at baseline (October 2013) and follow-up (March 2015).

More households at follow-up had used their smartphones or mobile devices to access the internet compared with baseline (64.8%, 129/199 vs 50.5%,101/199; *P*<.001; Figure 3). A total of 34 internet users who had never used a mobile device to access the internet at baseline reported using a mobile device for internet access at follow-up. Only 6 households reporting that they had stopped using a mobile device for internet access.

### Health-Related Internet Use

**Figure 4:**
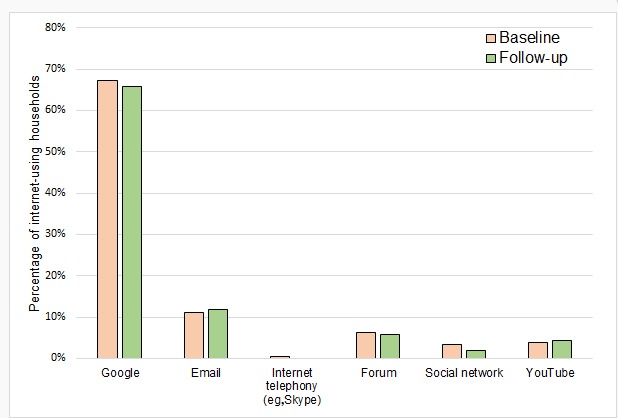
How internet-using households had used the internet for health-related activities.

Most internet-using households had used the internet for at least one health-related activity; there was no significant difference between baseline (70.2%, 144/205) and follow-up (68.7%, 145/211; *P*=.29) for health-related internet use (Figure 4). However, at follow-up, internet users showed a significant increase in self-reported confidence in using the internet for health-related tasks (mean=7.39 vs mean=7.78; *t*_197_=2.88; *P*=.004; CI=0.12 to 0.65).

No significant differences were found for the uses of the internet for health-related tasks between baseline and follow-up for internet-using households. The most common health-related activity was using a search engine to search for health topics (67% vs 66%), followed by using email for health (11% vs 12%) and discussing health topics on a forum (6% vs 6%).

Use of social media for health remained low; only 6 households (3%) at baseline and 4 at follow-up (2%) reported use.

### Self-Perceived Barriers

Just over half (54.0%, 128/237) reported that they “have or would use the internet for health and have no real barriers to that use.” There was no change at follow-up (58.6%, 139/237). The most common reported barrier at follow-up was “No need for health information” (11.0%, 27/246) and “I have no interest in using the internet” (11.0%, 27/246). Only 3 households (1.2%, 3/246) reported that they “Would use the internet more for health if I could get a good internet connection.”

**Table 1:** Summary of electronic health readiness and the 4 subvariables on matched households.

Variable	Baseline		Follow-up		Change	
	Mean	SD	Mean	SD	Mean	SD
Electronic health readiness	4.36	1.72	4.59	1.78	*0.23* ^a^	0.06
Personal	5.49	2.92	5.77	2.94	*0.28* ^a^	0.02
Provision	4.06	1.70	4.26	1.78	*0.20* ^a^	0.08
Support	1.89	1.87	1.88	1.80	−0.01	−0.07
Economic	1.57	0.90	1.67	0.88	0.10	−0.02

^a^Values in italics have *P*<.01.

### Electronic Health Readiness

Of the 236 households with complete data, half (51.3%, 121/236) showed no change in their eHealth readiness score, a third (33.5%, 79/236) showed an increase in their eHealth readiness score (maximum increase 3), and 36 (15.3%, 36/236) showed a decrease in their eHealth readiness score (maximum decrease of −3).

Overall eHealth readiness scores increased significantly from baseline to follow-up for these 236 households (mean=4.36 vs mean=4.59; *t*_235_=4.18; *P*<.001; CI=0.13 to 0.35). The SD of readiness (eHealth inequalities) among responders remained similar (1.72 vs 1.78). Analyses of the 4 subvariables that contribute to the calculation of eHealth readiness scores indicated that both personal and provision subvariables increased over the 18 months (*t*_255_=3.191; *P*=.002 and *t*_258_=3.410; *P*=.001), whereas economic (*P*=.12) and support (*P* *=*.97) subvariables showed no significant change (Table 1).

As might be expected, new internet users had higher increases in their readiness scores compared with continued users (1.56 vs 0.26; *t*_197_=−4.76; *P* ≤.001).

Previous users who had stopped using the internet (new nonusers) showed the biggest decreases in their readiness score, with an average reduction of 1.75. These 5 households had significantly lower readiness scores at baseline (3.00 vs 5.04; *t*_197_=3.78; *P*<.001) than the 195 who were *continued* *users*.

As new adopters of the internet showed the largest increase in readiness scores, potentially these households alone may have been responsible for the sample increase in readiness scores. To investigate this, further analysis was conducted on *continued* *users*, excluding new internet users; this showed significant increases in readiness scores (mean=5.04 vs mean=5.30; *t*_189_=4.57; *P*<.001; CI=0.15 to 0.38).

## Discussion

### Overall Impact

No one has previously examined the impact of concurrent improvements in internet infrastructure alongside person-based interventions. We assessed the impact of such interventions on personal eHealth readiness via a cluster quasi-randomized factorial controlled trial. Although eHealth readiness increased over the course of the study, this change could not be explained by the interventions, either singly or in combination. This could be because there really was no improvement or that the questionnaire approach we used was not sensitive to the change.

Our single booklet posted to a house and short limited discussions with a few practices was very “low dose.” The implementation of *Superfast* could potentially have more impact, but it relies on uptake. As a pragmatic randomized controlled trial, our analysis was *intention-to-treat*; thus, low uptake in all 3 arms could *swamp* a possible improvement that might be seen in an *as treated* analysis.

The use of eHealth relies on 3 separate but supporting conditions: (1) the personal ability to use it, (2) the presence of systems to provide it, and (3) the infrastructure available to support it. For this reason, despite finding no positive impact of any of the interventions, we argue that this study demonstrated a possible method to explore the impact of infrastructure improvements alongside complementary interventions.

### Changes Across Cornwall in Electronic Health Readiness and Electronic Health Inequalities

A third of our household respondents improved their eHealth readiness over 18 months of study, and overall, the mean eHealth readiness had improved without any increase in eHealth inequalities. There was no evidence that it was the already “eHealth ready” becoming further advantaged over the unconnected; people from across the whole “scale” had shown improvements.

The increased eHealth readiness reflected the increase in the proportion of internet users (79% vs 82%) in line with reported change for the United Kingdom [72]. Only 1 in 5 nonusers at baseline had started to use the internet, whereas only 5 people (<3%) had stopped using the internet at follow-up. But the overall increase in eHealth readiness was not solely due to new internet users. When new users were excluded from the analysis, the increase in readiness was still significant. This suggests that existing internet users became more ready to use the internet for health.

Despite the increase in the level of readiness to use eHealth, the types of use remained the same. Using a search engine to find health information was the most frequent activity. Although others have proposed that social media could be used more for health [73], few people in this study used it to obtain health information or contact health care professionals or organizations.

### Implementation of Superfast

Our *intention-to-treat* analysis examined the impact of *Superfast* regardless of whether a household adopted the service by upgrading their internet supply. On this basis, there was no improvement in readiness scores between early and late receivers of *Superfast* during the 18 months of study. However, at our study’s follow-up date, BT-estimated uptake of *Superfast* across Cornwall was still quite low (28%) despite *Superfast* being available for most of Cornwall. It was not possible to obtain data on which households in our sample had adopted *Superfast;* therefore, it was not possible to conduct an *as treated* analysis, that is, we cannot tell if households who adopted *Superfast* had higher readiness scores.

Analysis of categorical responses suggests more subtle changes. More late receivers than early receivers were happy with their internet speed at follow-up. As early receivers had become accustomed to the greater speed or bandwidth, both their internet use and expectations of broadband provision are likely to have increased.

Slow uptake of *Superfast* is also seen nationally; Ofcom [74] reported that by the end of 2015, only 42% of UK households had taken up offered *Superfast* services. This uptake was higher than Cornwall’s uptake, but *Superfast* had been available in other parts of the United Kingdom for longer. A total of 38% thought cost was a concern, which is quite a high proportion of people might be therefore put off by switching to superfast. We do not know if these people remained with slower speed access rather than take up superfast because of cost.

Only 3 respondents said they would use eHealth more if they could get a good internet connection, suggesting that very few perceived their current internet speed as a barrier to eHealth use. However, at the time of this study, no Web-based health services available in Cornwall required a S*uperfast* connection. The most common eHealth activity was using Google, and most of the Web-based health information was in simple text and picture format not requiring a high-speed connection. At the time of our data collection, very few (<5%) reported using health services requiring higher speeds, such as YouTube. Nationally, in 2016, the NHS YouTube channel had less than 26,000 subscribers. Moreover, even video streaming only required modest speed connections of around 500 Kbps [75].

*Superfast* rollout seemed to have had a measurable impact on people’s perception of internet speed, but this did not translate to measurable increases of eHealth readiness. On the other hand, Cornwall is now structurally more ready to adopt eHealth services such as video calling.

### Tailored Household Booklet

Our study demonstrated how TBs could be produced based on questionnaire and geography. For example, for internet nonusers, it provided guides on how to get started. For internet users, it provided information on how and where they could find and use Web-based health information.

Booklets included information about Web-based services provided by recipients’ local GP.

However, this 1 booklet delivered by post and then asked about some months later was not effective. The recollection of receiving a booklet was extremely low, with only 5 households reporting they had received a booklet. The most likely explanation is that the booklet was perceived as junk mail and never read. The booklet design may be of use for other organizations such as Healthwatch or if given directly by a GP or practice nurse, but as a low-intensity intervention delivered by hand to the house, it was ineffective.

### General Practice Intervention

The GPI was ineffective. Few GPs agreed to meet the lead author, and the discussions suggested that these GPs were knowledgeable about eHealth. For example, the researcher raised the potential use of a phone triage system, championed by some GPs [76], but many GPs responded mentioning a recent article in the *Lancet* [77] that had shown increased workloads. The likelihood, therefore, was that the researcher was no better informed than the GPs and, thus, was unlikely to raise awareness of new digital possibilities.

On the other hand, where the researcher attended larger practice meetings, it was apparent that the views and opinions of GPs differed drastically within the same surgery. In 1 meeting of note, the topic of information prescription sparked a huge debate over its usefulness. Several GPs within the practice were very positive toward information prescription, often directing patients to specific URLs with information on their condition and even printing out Web-based information for those who had limited access. Other GPs had very strong views against using information prescription, preferring that the patient spoke to them only and not use the internet. This discrepancy in GPs’ attitude has been well documented [78] and highlights the continued inequalities of service provision.

The difficulty in recruiting GPs has been demonstrated in previous research [79]. The GPI was designed to prevent this, by being short in length with minimal requirements for GP participation, although this did not seem effective. The time of year may have prevented a higher participation rate, with many GPs citing a busy flu season impacting their availability. However, it is likely that GPs will always be busy [80] and reluctant to participate, without a keen indication of potential benefits.

Addressing barriers to the implementation of eHealth technology is a complex process that requires support from health services. It is important for policy makers and hospital or practice managers to understand the specific barriers that challenge the practicing GPs and design appropriate interventions to address barriers and promote facilitating factors [81]. Some barriers such as cost associated with the adoption and maintenance of eHealth technology may require incentive [82].

### Limitations

The study suffered from a low response rate; responders were disproportionally female, older, and came from areas with higher estimated house values. As the *Superfast* rollout was outside the control of the researchers, it was not possible to randomize. Early receivers of *Superfast* were likely from areas in Cornwall that had existing internet infrastructure and were less rural. Therefore, it is possible that late receivers contained a higher proportion of isolated households. The sampling method, designed to reduce the potential of contamination between arms of the study, removed postcodes with shared GP practices following selection. As the selection of postcodes was ordered by population, a limited measure or rurality, postcodes that shared a GP practice with another more populated postcode had a much lower chance of being selected. Although this approach was vital to reduce the high risk of contamination between intervention conditions, it again meant that highly rural postcodes may have less chance of being included. It is possible, therefore, that the eHealth readiness from this sample is overoptimistic compared with Cornwall as a whole.

### Further Research

This study has provided 2 measurements of eHealth readiness within Cornwall over an 18-month period. There is potential to continue this study to provide a longitudinal view of the change in eHealth readiness over the coming years. A continued longitudinal study will provide insight into the change over time and allow for the impact of the *Superfast* rollout to be further assessed. As discussed, the actual uptake figures of *Superfast* are low, estimated at 28%, but these are expected to increase over the coming years. Continued measurement may show a continued increase in eHealth readiness as the uptake rates increase. Importantly, it will also allow for the inequalities in readiness to be monitored.

With the implementation of *Superfast* across the county, Cornwall has the potential to be a prime location for research into eHealth. The infrastructure improvement has made it possible for Cornwall to support highly demanding eHealth services such as video consultations or live streaming of health clinics. Presently, the county does not provide such systems, but now has the structural groundwork for research in this area. There is the potential for randomized trials of such services to be organized and conducted. Small trial projects might have to be conducted at the hospital level to show feasibility. This study will help show the potential benefits of such services, which may encourage innovations to be adopted more widely, such as at the GP level. In addition, future research in the area will provide further insight into the significant barriers toward eHealth use; with the *physical* speed barrier removed, other personal and organizational barriers are likely to be further highlighted. This will help researchers examine how to address those barriers and design effective interventions.

### Conclusions

Over the 18-month study, households in Cornwall became more *eHealth ready*. It is possible that the rollout of *Superfast* contributed to this, but we were unable to show that definitively. It is unlikely that our other 2 interventions had any effect. The study illustrates the complexity of trying to assess such interventions by a randomized trial, and our methods for a cluster quasi-randomized factorial controlled trial may be of use for others.

## Appendix

Multimedia Appendix 1 The modified Personal eHealth Readiness Questionnaire used to measure eHealth Readiness, which was delivered to participants at baseline and follow-up.

Multimedia Appendix 2 Example of the page database used to construct tailored booklets, which were delivered to all households within the tailored booklet intervention arm of the study.

## Abbreviations

BT: British Telecom
eHEALS: eHealth literacy scale
eHealth: electronic health
GP: general practitioner
GPI: general practice intervention
ICT: information and communication technology
NHS: National Health Service
NS: did not have Superfast
PERQ: Personal eHealth Readiness Questionnaire
S: Superfast
TB: tailored booklet
TBI: tailored booklet intervention
