# Supplementary material for: The Impact of Superfast Broadband, Tailored Booklets for Households, and Discussions With General Practitioners on Personal Electronic Health Readiness: Cluster Factorial Quasi-Randomized Control Trial
Source: J Med Internet Res. 2019 Mar 11;21(3):e11386. doi: 10.2196/11386 (PMC6431827; doi:10.2196/11386)
Supplement: Multimedia Appendix 1 [file jmir_v21i3e11386_app1.pdf]

The Impact of Superfast Broadband, Tailored Booklets for Households, and Discussions With General Practitioners on Personal Electronic Health Readiness: Cluster Factorial Quasi-Randomized Control Trial

Appendix 1

Philip Abbott-Garner, PhD	Postgraduate student
Janet Richardson, PhD	Professor Health Services Research
Ray B. Jones, PhD	Professor Health Informatics

All School of Nursing and Midwifery
Plymouth University

Correspondence to
Professor Ray Jones
School of Nursing and Midwifery
Plymouth University
Drake’s Circus
Plymouth PL4 8AA

Ray.jones@plymouth.ac.uk

Appendix 1 – The PERQ measure used in the study

PLEASE COMPLETE THIS GREEN QUESTIONNAIRE IF YOU ‘HAVE USED’ THE INTERNET IN THE PAST 3 MONTHS
ID=1000

A. INTERNET USE FOR ANY PURPOSE
This section is about whether you have used the Internet, how often and where you use it.

A1) Typically how often do you use the Internet for any purpose?

☐ Many times a day ☐ At least once a day

☐ At least once a week ☐ Less than once a week - every now and then

A2) What have you used the Internet for? *Tick boxes in the first column for all the ways you have used the Internet for any purpose, and tick boxes in the second column for all the ways you have used the Internet for something related to your health.*

	Have used the Internet for…..	
	Any Purpose	For something Health Related
To find information (e.g. using Google)		
Email		
Internet telephony (e.g. Skype)		
Discussion forum		
Twitter		
Social network site (e.g. Facebook, Linked in)		
Watching videos (e.g. YouTube)		
Online Gaming or Virtual World (e.g. World of Warcraft, Second Life)		

A3) Where and how have you accessed the Internet in the last 3 months? Tick all that apply

☐ Desktop / laptop computer at home ☐ Smart phone or mobile device (e.g. iPhone, iPad)

☐ Desktop / laptop computer at work ☐ Computer in a library or community centre

☐ ‘Paid for’ computer in an Internet café, shop, airport

☐ Elsewhere

B. ACCESS TO INTERNET SERVICES
This section is about your access to the Internet and the services available to you.

B1) Does your home (tick ✓ one)……..

☐ Have an Internet connection that is fast enough for what **you need** (Go to B3).

☐ Have an Internet connection that is slow for what **you need**

☐ Have no Internet connection

☐ Don't know

B2) If you do not have an Internet connection, or it is slow for what you need, why is that? (tick ✓ one)

☐ I would need to pay more

☐ I live in a rural area and there is no good connection to my home

☐ My local server is congested and unreliable

☐ My Internet provider does not offer a faster connection

☐ Don't know

B3) Does your General Practitioner (family doctor) have a website (e.g. that you might find by Google)?

☐ Yes I have looked at it ☐ Yes I think so but I have not seen it

☐ No ☐ Don't Know

If your General Practitioner (GP) has a website AND you have looked at it:

B4) If you wanted, can you order a repeat prescription by email, or on your GP's website?

☐ Yes, I **have** done so ☐ Yes, but I have **not** done so ☐ No ☐ Don't Know

B5) If you wanted, can book an appointment online to visit your GP?

☐ Yes, I **have** done so ☐ Yes, but I have **not** done so ☐ No ☐ Don't Know

B6) If you wanted, can you see your own medical record online via your GP's website?

☐ Yes, I **have** done so ☐ Yes, but I have **not** done so ☐ No ☐ Don't Know

B7) In the last three months, have you used the Internet trying to find information about **health** topics, services, treatments, advice etc?

☐ Not tried in the last 3 months ☐ Tried, and found what I wanted most of the time

☐ Tried, but not been able to find what I wanted

B8) In the last three months, have you used the Internet trying to contact an organisation online, or discussion forum, or other people, for some reasons connected with **your health** and been able to get what you wanted?

☐ Not tried in the last 3 months ☐ Tried and found what I wanted most of the time

☐ Tried, but not been able to contact who I wanted

C. PERSONAL SKILLS, CONFIDENCE, AND SUPPORT IN USING THE INTERNET FOR HEALTH

This section is about your skills in using the Internet and if you could, or have, given support to others in using the Internet for health.

C1) Do you have a long term disability that makes using the Internet difficult?

☐ No

☐ Yes, makes using the Internet very difficult

☐ Yes, makes using the Internet somewhat difficult

C2) This question is a self-assessment of your Internet skills, not necessarily concerned with health. In the following table, please read the 'task' and then tick one box to show if you think you could do that task.

I think I could…..	No	Maybe	Yes
Book tickets for a film online and save a copy of the booking into a folder on your computer			
Go online to check symptom information from a trusted health source			
Use Google to find out what type of documents you need to apply for a new passport if yours was lost			
Locate healthcare facilities in my local area, then plan how to travel there			
Create an online social media page, upload photos into an album and comment on the photos			
Visit a forum to discuss health issues with others and/or professionals			

C3) In general how confident are you in using the Internet for **health related tasks**?

Circle a number between 1 (not at all confident) and 10 (totally confident).

Not confident…………………………………………………………….Totally confident									
1	2	3	4	5	6	7	8	9	10

C4) In the past 6 months have you received a leaflet in the post regarding using the **Internet for health?**

☐ Yes ☐ No ☐ Don't Know

C5) Has a doctor, nurse, or other health professional ever given you information (e.g. a web address) to help you use the Internet for your health?

☐ Yes ☐ No ☐ Don't Know

C6) If you, or someone in your household, wanted help using the Internet, could you find it near where you live, or by phone or email? (e.g. from local library, Age UK, local authority, NHS, or University).

☐ Not that I'm aware of ☐ Don't Know

☐ Yes

If yes, have you ever made use of such help? ☐ Yes ☐ No

C7) Would you be willing to help a person in your local community use the Internet for their or their family's health by offering training or support?

☐ Yes ☐ Yes but only over phone or email

☐ No I don't think I have the skill to help ☐ No

C8) Have there ever been times when help from somebody in using the Internet for your or your family's health was or might have been useful for you?

☐ Yes ☐ No

If NO to C8, go to section D

C9) Do you have a family member or friend who could help you to use the Internet (for any purpose)?

☐ No ☐ Yes, there is someone I can ask quite easily

☐ Yes, but they are not, or would not be very easy to ask

C10) **If yes to question C9,** would you feel OK about asking them to help you use the Internet for your or your family's **health** purposes (to find information or to communicate with someone)?

☐ Yes

☐ No

D. ECONOMIC CONSIDERATIONS

This section asks about the cost to you of using the Internet for health or of accessing health services.

D1) To get an idea of comparative costs, please read each of the statements and tick one box for each to show whether you agree or disagree, as they relate to you at the moment.

For me………	Strongly agree	Agree	Disagree	Strongly disagree	Don't know
The monthly cost of home Internet is a major concern					
Mobile Internet access on smart phones and tablets (e.g. iPad) is expensive					
Getting to a public library to use the Internet does not cost much					
It costs me nothing, or very little, to get to see my GP					
It costs me nothing, or very little, to visit my nearest hospital					

E. OVERALL VIEWS ABOUT USING THE INTERNET FOR HEALTH

This section asks about the factors most likely to reduce your use of the Internet for health, and for your views.

E1) Which **one** statement best sums up how you feel about using the Internet for health? None of them may be exactly right, but try to choose one and then you can further explain your answer in the space in E2.

☐ I have no need for health information

☐ I don't understand the Internet that much

☐ I would use the Internet more for health if I could get a good Internet connection

☐ I would use the Internet more for health if more online health services were available to me

☐ I would use the Internet more for health if I could get someone to help me

☐ I would use the Internet more for health if money were no object

☐ I have or would use the Internet for health and have no real barriers to that use

E2) Do you have any thoughts about using the Internet for health? What could be done to help those who want access to the Internet for health? (Also use this space if you want to further explain your answer to E1).

……

……

……

F. ABOUT YOU, HEALTH INFORMATION AND SUPPORT
This last section asks for information about you. Please note that all information will be held confidentially and securely.

F1) First Name: .... Last Name: ....

F2) Gender: ☐ Male ☐ Female

F3) Age: ☐ 16-24 ☐ 25-34 ☐ 35-44 ☐ 45-54 ☐ 55-64 ☐ 65-74 ☐ 75+

F4) As an estimate how far is it to your GP? (1 mile = 1.6km)

☐ Less than 1 mile ☐ 1 – 3 Miles ☐ 3 – 5 Miles ☐ 5 – 10 Miles ☐ 10+ Miles

F5) How do you usually travel to your GP Surgery? (tick one only)

☐ Walk or cycle ☐ Public Transport ☐ Lift from friends or family in their vehicle

☐ Drive in own vehicle ☐ Taxi ☐ Other

F6) As an estimate how many times have you visited your GP in the past year? ....

F7) Have you had any hospital appointments in the last year?

☐ Yes ☐ No

F8) If yes, how many?............

F9) How would you normally travel to hospital?

☐ Walk or cycle ☐ Public transport ☐ Lift from friends or family in their vehicle

☐ Drive in own vehicle ☐ Taxi ☐ Hospital arranged transport

☐ Other

F10) In the last three months have you (tick ✓all that apply):

Seen a doctor, nurse, or other health professional about your health	
Asked a family member or friend something about your health	
Phoned a helpline (e.g. NHS Direct, Samaritans, Diabetes UK) about your health	
Read a book, or magazine to find something out about your health	
Used the Internet for something to do with your health	
None of the above	

Thank you very much for taking time to complete the questionnaire.
Please return in the prepaid envelope.

PLEASE COMPLETE THIS PINK QUESTIONNAIRE IF YOU ‘<u>HAVE NOT USED</u>’ THE INTERNET IN THE PAST 3 MONTHS

A. FOR PEOPLE WHO HAVE NOT USED THE INTERNET IN THE LAST THREE MONTHS

This section asks about if you have EVER used it, whether you would like to use the Internet, if maybe you would like to use it for health related things, given help.

A1) Have you EVER used the Internet (for any purpose)? (Tick one of the following)

- ☐ I used to use it fairly often but not recently
- ☐ I have only ever used it a few times and not recently
- ☐ I have never used it

A2) Do you have a long term disability that would make using a computer difficult?

- ☐ No
- ☐ Yes, makes using the internet very difficult
- ☐ Yes, makes using the internet somewhat difficult

A3) Does your home have an Internet connected computer? ☐ Yes ☐ No

A4) As far as you know do any of your neighbours have Internet access?

☐ Yes ☐ No ☐ Don’t Know

A5) Has anyone ever used the Internet <u>for you</u> (e.g. to find out something for you, or to buy something for you, or to contact someone on your behalf by email)?

☐ Yes ☐ No

A6) If someone was able to help you, would you ‘have a go’ at using the Internet?

☐ Yes ☐ Probably ☐ Possibly ☐ No, it’s not really for me

A7) If you would ‘have a go’ using the Internet, do you have someone (e.g. family, friend, neighbour) who could help you?

- ☐ Yes, there is someone I can ask quite easily
- ☐ No
- ☐ Yes, but they are not, or would not be very easy to ask

A8) If someone was able to help you, <u>and</u> it was easy, <u>and</u> it was cheap, would you use a home Internet connection?

☐ Yes ☐ Probably ☐ Possibly ☐ No

A9) If there were Internet connected computers available at some place (such as the local library) that you go to, and they were free to use, easy to use, and there was help there to use them for any purpose, would you consider using them?

☐ Yes ☐ Probably ☐ Possibly ☐ No

B. ECONOMIC CONSIDERATIONS
This section asks about the cost to you of using the Internet for health or of accessing health services.

B1) To get an idea of comparative costs, please read each of the statements and tick one box for each to show whether you agree or disagree, as they relate to you at the moment.

For me………	Strongly agree	Agree	Disagree	Strongly disagree	Don't know
The monthly cost of home Internet is a major concern					
Mobile Internet access on smart phones and tablets (e.g. iPad) is expensive					
Getting to a public library to use the Internet does not cost much					
It costs me nothing, or very little, to get to see my GP					
It costs me nothing, or very little, to visit my nearest hospital					

C. OVERALL VIEWS ABOUT USING THE INTERNET FOR HEALTH
This section asks about the factors most likely to reduce your use of the Internet for health, and for your views.

C1) Which **one** statement best sums up how you feel about using the Internet for health? None of them may be exactly right, but try to choose one and then you can further explain your answer in the space in C2.

- ☐ I have no need for health information
- ☐ I have no interest in using the Internet
- ☐ I would use the Internet more for health if I could get a good Internet connection
- ☐ I don't understand the Internet that much
- ☐ I would use the Internet more for health if I could get someone to help me
- ☐ I would use the Internet more for health if money were no object
- ☐ I would use the Internet more for health if more online health services were available to me
- ☐ I have or would use the Internet for health and have no real barriers to that use

C2) Do you have any thoughts about using the Internet for health? What could be done to help those who want access to the Internet for health? (Also use this space if you want to further explain your answer to C1).

……………………………………………………………………………………………………

……………………………………………………………………………………………………

……………………………………………………………………………………………………

D. ABOUT YOU, HEALTH INFORMATION AND SUPPORT
This section asks for information about you. Please note that all information will be held confidentially and securely.

D1) First Name: .... Last Name: ....

D2) Gender: ☐ Male ☐ Female

D3) Age: ☐ 16-24 ☐ 25-34 ☐ 35-44 ☐ 45-54 ☐ 55-64 ☐ 65-74 ☐ 75+

D4) As an estimate how far is it to your GP? (1 mile = 1.6km)

☐ Less than 1 mile ☐ 1 – 3 Miles ☐ 3 – 5 Miles ☐ 5 – 10 Miles ☐ 10+ Miles

D5) How do you usually travel to your GP Surgery? (tick one only)

☐ Walk or cycle ☐ Public Transport ☐ Lift from friends or family in their vehicle

☐ Drive in own vehicle ☐ Taxi ☐ Other

D6) As an estimate how many times have you visited your GP in the past year? ....

D7) Have you had any hospital appointments in the last year?

☐ Yes ☐ No

D8) If yes, how many?............

D9) How would you normally travel to hospital?

☐ Walk or cycle ☐ Public transport ☐ Lift from friends or family in their vehicle

☐ Drive in own vehicle ☐ Taxi ☐ Hospital arranged transport

☐ Other

D10) In the last three months have you (tick ✓all that apply):

Seen a doctor, nurse, or other health professional about your health	
Asked a family member or friend something about your health	
Phoned a helpline (e.g. NHS Direct, Samaritans, Diabetes UK) about your health	
Read a book, or magazine to find something out about your health	
Used the Internet for something to do with your health	
None of the above	

Thank you very much for taking time to complete the questionnaire.
Please return in the prepaid envelope.
