# Supplementary material for: The Impact of Superfast Broadband, Tailored Booklets for Households, and Discussions With General Practitioners on Personal Electronic Health Readiness: Cluster Factorial Quasi-Randomized Control Trial
Source: J Med Internet Res. 2019 Mar 11;21(3):e11386. doi: 10.2196/11386 (PMC6431827; doi:10.2196/11386)
Supplement: Multimedia Appendix 2 [file jmir_v21i3e11386_app2.pdf]

The Impact of Superfast Broadband, Tailored Booklets for Households, and Discussions With General Practitioners on Personal Electronic Health Readiness: Cluster Factorial Quasi-Randomized Control Trial

Appendix 2

Appendix 2. Pages Database

BRANDIS CORNER WITH PLYMOUTH UNIVERSITY

Philip Abbott-Garner
Plymouth University
Faculty of Health & Human Sciences
Room 103
4 Portland Villas
PL4 8AA
philip.abbott-garner@plymouth.ac.uk
Telephone: 01752 586570

Dear Occupant,

This booklet has been designed to make you aware of both local and national health services which may be of interest and use to you.

The enclosed information has been checked to ensure that it is both authentic and credible.

It is designed for internet and non-internet users.

If you have any enquires or comments regarding the booklet please feel free to contact me using the details listed at the top of the page.

Yours Sincerely,

Philip Abbott-Garner

PhD Student
Plymouth University

Using the Internet for Health

There are lots of good quality sites for information on the Internet including sites such as Health Talk Online, Dementia UK, British Heart Foundation, Alzheimer's Society, Mind, Stroke Association, Macmillan Cancer Support.

Most of these good quality sites are run either by the **NHS** or a **charity**.

There are of course lots of other sites offering health information from individuals, groups or commercial companies however this information may not be accurate or come from a reliable source. When it comes to your health it is best to stick to well respected sources such as those mentioned above.

NHS Choices is a good gateway to reliable and trusted health advice which has been certified by The Information Standards agency.

Don't forget that the Internet is **MORE** than just information, there are lots of discussion forums such as those that can be found on 'Patient.co.uk' where you can get advice and support from others with similar conditions.

Some people chat about their health conditions on social media including Facebook and Twitter. That's a possibility too, but you should remember that your posts may be public so you will want to consider carefully what information you choose to disclose.

There are also opportunities for you to see what other people think of local services or for you to feedback your ideas via Patient Opinion or Patient Choice.

The web addresses for the discussed websites can be found at the back of this booklet.

NHS choices
your health, your choices

www.nhs.uk

What is NHS Choices?

The NHS Choices website is a great way to find health information, provide feedback and hear about other people's health experiences.

There's loads of advice about medicine and symptoms. The website can also help you make other important decisions about your health.

Why is NHS Choices useful?

Being healthy is important. The NHS Choices website has easy to follow, reliable and trusted advice, bringing together expert information from across the NHS.

What's more, because it's online, this resource is at your fingertips 24/7, it's completely free and up-to-date.

Where can I find the information I need?

**Browse Health A-Z**	www.nhs.uk/Conditions
**Check your symptoms**	www.nhs.uk/SymptomCheckers
**Find out more about medicine**	www.nhs.uk/medicine-guides
**Read common health questions**	www.nhs.uk/chq
**Find a service near you**	www.nhs.uk/Service-Search

information prescription service

www.nhs.uk/IPG

What is an information prescription?

Patients and carers want to know how to find information they can trust and rely on. Information prescriptions (IP) are a quick and easy way to find about your condition and local services.

Information prescriptions bring together a wealth of information sources from NHS Choices and charity partners to make it easier for you to find the information you need.

Creating an information prescription

1) Visit **www.nhs.uk/IPG**
2) Enter the condition you would like to know more about and your postcode (optional)
3) You can then select from a variety of content (including text, video, audio) from several sources.
4) After selecting the desired content click 'Create Information Prescription'.
5) Finally you can choose to email, print (as a PDF) or save the IP by clicking the corresponding button at the top of the screen.

More information can be found at:

www.nhs.uk/IPG/Pages/AboutThisService.aspx

Health information on Social Media

Social media has taken the world by storm. It's much more than a way to share painfully adorable pictures of kittens, it's also a way of finding and sharing valuable information.

The NHS is increasingly using Social Media to engage with patients and provide up to date information.

Below is a list of the NHS presence on Social Media. Please remember that your posts may be public so you will want to consider carefully what information you choose to disclose.

www.youtube.com/NHSChoices

The service is intended to help you make choices about your health, from lifestyle decisions about things like smoking, drinking and exercise, through to the practical aspects of finding and using NHS services in England when you need them.

@NHSChoices
twitter.com/NHSChoices

Official site of the NHS England. Everything health, including: Health A-Z, lifestyle advice, performance data & patient ratings. Monitored weekdays 9-5.30.

www.facebook.com/NHSChoices

The NHS facebook page keeps you updated on the latest health news and provides a community to help you improve your health. They post regularly about getting fit, losing weight, improving your mental wellbeing and many other popular topics.

The page aims to bring you trusted information and advice to help you lead a healthier lifestyle. It is also a place for you to share your own experiences and discuss health topics with others.

Health apps for your phone or tablet

NHS choices health apps library

apps.nhs.uk

Safe and **trusted** apps to help you manage your health

- Reviewed by the NHS to ensure they are clinically safe and relevant to people living in England.
- Rated by you and the health care community.
- Categories include: Conditions, Healthy living, Health information & Social Care.

Some Examples

British Heart Foundation Recipe Finder

Great meal options for people with cholesterol, high blood pressure and/or diabetes.

Healthy Living App

Healthy Living application is the most comprehensive guide on healthy living, designed with all of us in mind: the young, the old, the busy on the go people. Filled with useful information on health tips, help and advice

Additional online health services

**Royal Cornwall Hospitals Trust**	www.rcht.nhs.uk
**Macmillan Cancer Support**	www.macmillan.org.uk
**Mind**	www.mind.org.uk
**Stroke Association**	www.stroke.org.uk
**Alzheimer's Society**	www.alzheimers.org.uk
**Health Talk Online**	www.healthtalkonline.org
**Dementia UK**	www.dementiauk.org
**British Heart Foundation**	www.bhf.org.uk
**Patient.co.uk Discussion Forums**	www.patient.co.uk/forums
**Patient Opinion**	www.patientopinion.org.uk
**NHS Patient Choice**	www.nhs.uk/comment

GPs close to your location

Your local GPs website can offer useful services to help you save time and easier manage your healthcare.

Online Repeat Prescription

This services make it more convenient for you to manage your repeat prescriptions saving you time. Prescriptions can be ordered online and collected from your local pharmacy without the added hassle of contacting your GP practice.

Online Appointment Booking

This allows you to book an appointment with your GP using their website. GP phone lines can often be busy making it difficult to get through and requiring multiple phone calls. By booking online you can reduce hassle and potentially arrange your appointment faster.

Online Access to Medical Records

This allows you to view your medical record over the internet. It allows you to easily and quickly view the electronic medical information held about you in the GP system.

Below is a list of GPs within your area and the online services that are available through their website.

	Online Repeat Prescription	Online Appointment Booking	Online Access to Medical Record
**Blake House Surgery**<br>www.blakehousesurgery.co.uk<br><br>Bowhay Close<br>Black Torrington<br>Beaworthy<br>EX21 5QE<br>Tel: 01409335830	YES	YES	NO
**Shebbear Surgery**<br>www.shebbearsurgery.co.uk<br><br>Shebbear Surgery<br>Beech House<br>Shebbear<br>EX21 5RU<br>Tel: 01409 281221	YES	NO	NO

Like to help someone to use the internet?

www.digitalskills.com

digitalskills.com aims to help make the UK the world's most digitally skilled nation.

They are scouring the web for great resources for teaching digital skills and providing a space where anyone can ask and answer questions.

Their map is starting to collect all the info about Wi-Fi hotspots, projects, organisations and more.

How you can help

Join **digitalskills.com** and spread the word.

If you know of great resources you can tell the organisation.

If you have skills to share, please add yourself or your project to the map

If there's something they can build for the site, they would like to hear

Christine's Story - Staying connected to my Grandson

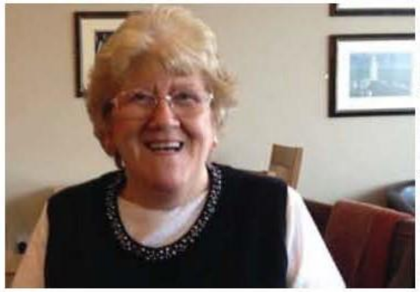

Christine Moore, aged 70, had rejected technology for more than 20 years, even leaving a job when computers were brought in. However, when her grandson travelled abroad to study, she soon discovered the internet would help her keep in touch and avoiding computers was no longer an option.

"My grandson Sam was at university and as part of his degree he was going to a college in America for a year. We've always been close and the idea of not seeing him for a year was terrible. My family started talking about how they'd be able to 'Skype' him to keep in touch but I had no idea what they were talking about!

"They showed me their tablet though, and explained how they'd be able to talk to him, and SEE him and I thought it was wonderful! I went straight out and bought my own tablet so I'd be able to keep in touch with Sam myself. It was a lot of money but I knew it would be worth it to see him.

"It was actually my 10 year old granddaughter Sarah who showed me how to get onto Skype and ring Sam. It was absolutely wonderful, the picture and sound was so clear it was like he was in the room!.

"Being able to keep in touch with Sam - and Sarah when she eventually goes off to uni or traveling - means the absolute world. I always thought technology wasn't for me, even if it meant leaving a job, but now I don't know how I'd manage without it. I only wish I'd tried it years ago!"

How have others benefited?

Plymouth SeniorNet, a project helping adults aged 65+ online, asked users how they benefited from using the internet.

**Better communication** with family, friends, or others by email or Skype, or being able to receive photos, or share things with your family or friends.	1st
**Being entertained or stimulated** by finding out facts, or playing games, having access to entertainment online, watching TV on 'catch up', taking part in hobbies, finding out about your family tree.	2nd
**Feeling more confident** because of your new skills and because you have a better idea of what is going on via the Internet and what things are happening.	3rd
**Being more independent** by being able to do something that otherwise someone may have had to help you with, or do for you. i.e. Internet banking, or applying for benefits online, or getting shopping delivered, or finding out about things.	4th
**Saving money or having a better range of goods** by shopping online or by finding out about prices and services online, for example, buying books or insurance online.	5th
**Better health care** because of things you have learned online, or through having better access to some online health services like online appointment booking, and prescriptions.	6th

Free training and assistance to use the Internet

UK onlinecentres™

Take your first steps online with UK online

Do more online!

There are so many things you can do on the internet. It could make everyday tasks a lot easier, save you time and money and allow you to do more of the things you like doing best!

It can help you get in touch with people wherever they are in the world at the click of a button, and you can find information on whatever you're interested in, from fishing to football.

Millions of people use the internet every day as it helps make their life easier – and you could join them!

Find the help and support you need

Whether you've never touched a computer before and want to get started with the basics, or can do a little but want to know more, we're here to help you.

Our friendly tutors are on hand whatever you want to learn about – from using a mouse for the first time to finding jobs online or keeping in touch with friends and family. We'll give you a helping hand so you're confident doing whatever you want to do online.

Places that offer help to use the Internet

UK online centres™

Your local UK Online Centre can help by providing access and training to use the Internet.

Listed below is your closest UKOC centre and your closest specialist home access centre.

Alternatively you can call: **0800 77 1234** to be directed to your nearest UK online centre for a free or low cost computer course.

Holsworthy Library

Partner Centre

At a Partner centre, you'll be able to find friendly help and support to improve your skills, as well as access to the internet

North Road,
Holsworthy
Devon
EX22 6HA
Tel: 01409 253514
www.devon.gov.uk/libraries

Opening Times:
Monday 14:00:00 - 19:00:00
Wednesday 10:00:00 - 19:00:00
Thursday 10:00:00 - 17:00:00
Friday 14:00:00 - 17:00:00
Saturday 10:00:00 - 13:00:00

Questions & Answers CIC

Specialist Centre - Home Access

These centres can offer support and advice to help you get online access in your own home, and may have a range of internet-enabled devices for you to try out.

4 Station Road,
Redruth,
Cornwall
TR15 2AB
Tel. 01209 200583
www.qacic.co.uk
james@qacic.co.uk

Opening Times:
Monday 09:30:00 - 14:30:00
Wednesday 09:30:00 - 14:30:00

Online learning to improve internet skills

learn my way – making online learning easy

www.learnmyway.com

What is Learn My Way?

Learn My Way is an online learning platform, built especially to make getting online easy. Learners can try the free online courses at home and work their way through with a friend or family member if they wish.

If you've got the basic online skills under your belt, this is the place to start learning a little bit more and increase your confidence in using the internet. Whether you want to learn how to shop online, access public services, complete online forms or learn how to use Facebook, there is a free online course to help you do it.

A full list of courses can be found here:

www.learnmyway.com/learn-more

"Even once you've got the basic online skills, there's still plenty in there to learn! I do all my shopping and banking online now. I'm still learning - I don't think I'll ever stop - but I'm not afraid of the computer anymore. Once you've made that first step, the world really is your oyster.""

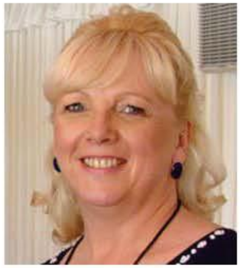

Karen, Kensington

Online learning to improve internet health skills

learn my way
making online learning easy

www.learnmyway.com/what-next/health

What is the Learn My Way Health Course?

Learn My Way is an online learning platform, built especially to make getting online easy. Learners can try the free online courses at home and work their way through with a friend or family member if they wish.

The Health course is a step by step online guide which will help you find out:

- How to use the NHS Choices website to look up conditions and illnesses
- What help is online to help you live a healthy life
- How to find and feedback on health services near you

The course is designed to help you make the most of the NHS Choices website.

"I've seen learners use the Health Resources on Learn my way to help them do everything from check symptoms when feeling poorly to help them stop smoking and check the rating of a hospital where they were about to have an operation! To see them getting healthier because of the help we provide getting them

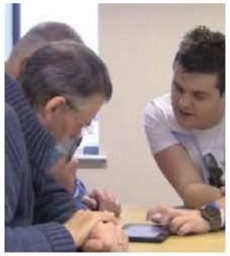

Help using the Internet with a disability

AbilityNet
Adapting Technology ▪ Changing Lives

Free IT support for disabled people

Reliable access to computers and the internet can change the lives of disabled people. Some people need specialist equipment but many use standard computer equipment, or new technology such as tablets and mobile phones. Some need help to choose the right equipment, others want to know more about the settings that are built into their computers.

That's why AbilityNet offers free services to people with a disability - They aim to help you get the most from computers and the internet.

Call their friendly team for any advice about computers and disability. If they don't know the answer they will know someone who does. It's a free service for disabled people and the families, friends and carers who support them.

Free helpline: 0800 269 545

www.abilitynet.org.uk

Thank you for taking the time to look through the booklet.

I hope the information within has been useful to you.

We welcome any enquires or comments regarding the booklet, please feel free to contact me using the details below.

Philip Abbott-Garner
Plymouth University
Faculty of Health & Human Sciences
Room 103
4 Portland Villas
PL4 8AA
Email: philip.abbott-garner@plymouth.ac.uk
Telephone: 01752 586570
